# Information, Advocacy and Signposting as a Low-Level Support for Adults with High-Functioning Autism Spectrum Disorder: An Example from the UK

**DOI:** 10.1007/s10803-017-3331-x

**Published:** 2017-10-23

**Authors:** Kris Southby, Olivia Robinson

**Affiliations:** 1 Centre for Health Promotion Research, Leeds Beckett University, Rm. 512 Calverley Building, City Campus, Leeds, LS1 3HE UK; 2 Institute for Health and Wellbeing, Leeds Beckett University, Leeds, UK

**Keywords:** Autism spectrum disorder, Adults, High functioning, Health and social care, Support

## Abstract

‘Low-level’ support is championed to support adults with high functioning autism spectrum disorder (HFASD) to achieve good quality health and social care, yet research in the area is sparse. Drawing on semi-structured interview data, this paper considers the efficacy of an intervention to provide low-level support to adults with HFASD with little or no funded support. The intervention led to a number of perceived positive outcomes for adults with HFASD, their families, and service providers in the city, including increased access to education, volunteering, support and information, socialising, improved health and wellbeing, and managing day-to-day. Although many of life’s difficulties still persisted, the intervention helped service users overcome barriers to availing further support, possibly leading to beneficial outcomes down the line.

## Introduction

Adults with autism spectrum disorder (ASD) who do not have an associated learning disability—so called ‘high functioning’ individuals (Lorenc et al. 2016)—are at risk of experiencing negative outcomes across a number of domains of life and to “function well below the potential implied by their normal range intellect” (Marriage et al. 2009). Adults with high functioning autism spectrum disorder (HFASD) have been found to experience poor and comorbid physical and mental health issues (Mazurek and Kanne 2010; Hofvander et al. 2009; Howlin and Moss 2012; Lake et al. 2014; Lugnegård et al. 2011; Mattila et al. 2010; Gotham et al. 2015); high unemployment, underemployment, and workplace discrimination (Baldwin and Costley 2016; Gotham et al. 2015; Howlin and Moss 2012; Smith et al. 2012; Taylor and Seltzer 2011; Lake et al. 2014); limited meaningful social and romantic relationships (Baron-Cohen and Wheelwright 2003; Farley et al. 2009; Jennes-Coussens et al. 2006; Tobin et al. 2014); and struggle to gain autonomy and independence in their lives (Renty and Roeyers 2006; Lake et al. 2014; Howlin and Moss 2012). Considering that just over 1% (and rising) of populations in the United Kingdom (UK) and the United States of America (US) are thought to have ASD (Autism and Developmental Disabilities Monitoring Network Surveillance 2010; Brugha et al. 2012) and that around 50% of these people are ‘high functioning’ (The National Autistic Society 2016), the negative outcomes experienced by adults with HFASD are likely to occur in large numbers with significant costs to individuals and society (Nicolaidis et al. 2013; Vogan et al. 2016) and need to be addressed.

There is a need for appropriate support to be in place to improve the quality of life for adults with HFASD (Baldwin and Costley 2016; Engström et al. 2003). Indeed, the availability of suitable support has been found to be a better predictor of quality of life for adults with HFASD than individual impairment (Kamio et al. 2013; Renty and Roeyers 2006). Policy aims in Western countries to provide good quality health and social care for adults with HFASD (i.e. Mental Health Commission of Canada 2012; National Institute of Mental Health 2016; The Westminster Commission on Autism 2016) are therefore to be welcomed. In the UK, the Autism Strategy for England (Department of Health 2010), the Autism Act 2009, and the Care Act 2014 purport to move services away from just crisis management towards preventative services and support for adults with ASD living in their communities. The most recent iteration of the Autism Strategy (Department of Health 2014) stresses the importance of ‘low-level’ support services based around interpersonal support networks and advice and information. Such support should be available to all adults with ASD “regardless of whether they are eligible for social care” or not (Department of Health 2015).

The aim of this paper is to consider the efficacy of providing low-level support in the form of information and signposting, advocacy, and mentoring to adults with HFASD. The paper adds to a limited body of existing research reporting the positive outcomes associated with low-level support for adults with HFASD. Such interventions would appear useful in fulfilling government aspirations of providing good quality health and social care for all adults with ASD.

### Low Level Support

Low-level support broadly concerns any non-intensive service aiming to provide general support, which is not directed at treating a clinical problem or deficit, to people in their everyday lives (Lorenc et al. 2016). The focus of low-level support can be wide ranging and can be delivered through a variety of settings, such as health services, social care, the community, and telephone and internet based services (Lorenc et al. 2016).

Previous research has identified positive outcomes for adults with HFASD from different low-level supports across different domains. Supported employment schemes have enabled individuals to find and maintain employment (Mawhood and Howlin 1999; West et al. 2013; Burke et al. 2010; Lawer et al. 2009). Both formal and informal support groups have been shown to benefit adults with HFASD in terms of increasing peer relationships, social skills, decision-making, and problem solving (Jantz 2011; Hillier et al. 2007; Howlin and Yates 1999; Tobin et al. 2014). Social-skills training has been found to increase knowledge of social skills and social functioning, and decrease loneliness (Tobin et al. 2014; Gantman et al. 2012; Palmen et al. 2012; Turner-Brown et al. 2008).

As well as low-level interventions that aim to support adults with HFASD in one domain of life or to achieve one specified outcome (i.e. employment, acquisition of a skill), a smaller number of interventions aim to provide more holistic support. The autism ‘one-stop-shops’ in Scotland, for example, are staffed and accessible premises that house services for adults with HFASD and provide information about, and signposting to, further support and services available elsewhere. Evaluation of sites in Edinburgh and Glasgow demonstrates the positive impact of autism one-stop-shops, “improving the social, physical and emotion wellbeing of adults with autism and building their capacity to live full and independent lives in the community” (Tait et al. 2013; National Centre for Autism Studies 2006, 2007).

A recent systematic review, however, of the current state of knowledge concluded that there is little or no evidence relating to many low-level strategies for supporting adults with HFASD (Lorenc et al. 2016). As such, this paper will contribute to the existing literature regarding the efficacy of low-level supports for adults with HFASD.

### About the Intervention

The research is based around the Leeds Autism AIM (Advocacy, Information, and Mentoring) service (‘the service’), which provides low-level support to adults with HFASD with little or no funded support, their families and carers, and other autism services in Leeds, England. The service consists of three overlapping core activities, all free at the point of use. Firstly, two weekly drop-in ‘autism hubs’—one in the city-centre, one in the south of the city—provide access to a range of support and information, café areas, quiet areas, computer rooms, and meeting rooms for adults with HFASD, their families/carers, and service providers. The drop-in hubs also host external support services, including advisors from the Citizens Advise Bureau and Department of Work and Pensions, which users can access. Secondly, ‘autism mentoring’ pairs individual service users with a specially trained volunteer for one-to-one support. Drop-in hub staff and mentors also act as advocates on behalf of adults with FHASD. Thirdly, ‘information and signposting’ provides information about, and signposts people to, other services in the region. Information is available in print and by speaking to staff at the drop-in hubs, on the Leeds Autism Directory website, and via telephone and email correspondence with the service.

A small staff team delivers the service. A project coordinator/manager has overall responsibility. A part-time volunteer coordinator oversees the recruitment and training of volunteers, who themselves support at the drop-in hubs or act as mentors. A part-time information officer is responsible for producing written materials and managing the website. Two support workers are also funded by the local authority to staff the drop-in hubs. Adults with HFASD are involved in the service steering-group and operationally involved as drop-in hub volunteers, peer-mentors, or in the delivery of group sessions at the drop-in hubs. The service was initially established in January 2015 with one drop-in hub (city centre), mentoring, and the information and advice service. The second drop-in hub was set up in the south of the city in March 2016.

Since inception, service use has gradually and consistently increased. The mean weekly attendance at the city centred drop-in hub has increased from 12.5 (including 2.5 new users) to 28.75 (including 4.5 new users) by September 2016, and the mean weekly attendance to the south Leeds hub has increased from 4 to 8.7 by September 2016. Between May 2015 and October 2016, the Leeds Autism Directory website had an average of 559 visits per month—‘employment’ was the most viewed section of the website. Since the mentoring scheme began, 29 people have been trained as mentors and 24 mentoring matches made. The mentoring services has been consistently oversubscribed.

## Methodology

The intention of data collection was to explore the perceived outcomes and impact of the service as well as key delivery mechanisms and areas of improvement. A qualitative methodology was adopted; drawing on qualitative data allowed a rich understanding of the lived experience of the service to gathered.

Semi-structured interviews were the main data collection tool and illuminated the ‘lived experience’ (Bryman 2016) of the service. Interviews were conducted in a range of different formats to suit the preferences of participants, including face-to-face at the city centre drop-in hub, via email, and over the telephone. Interviews lasted between 12 and 58 min (mean = 22 min). Participants were asked about their experience of the service, what outcomes they had achieved through their involvement with the service, and how the service could be improved. With permission from participants, interviews were audio recorded and transcribed.

### Sample and Recruitment

Given that adults with ASD and their families are the de facto experts of their needs, problems, and priorities (Gotham et al. 2015) interview recruitment was weighted in favour of adults with HFASD. An invitation to participate was emailed by the service manager to everyone on the service’s mailing list and a member of the research team attended the city-centre drop-in hub on four occasions, inviting those in attendance to be interviewed. The research team also purposively sampled relevant professionals connected to the service, including service staff, commissioners, and local authority Adult Social Care staff. Thirty interviews were conducted in total, including with adults with HFASD (n = 14), family members (n = 3), volunteer mentors (n = 2), and ‘professionals’ (n = 11). Following Lorenc et al’s (2016) call for research to reflect the diversity of ASD, the sample of adults with HFASD involved was heterogeneous in terms of age, employment status, involvement with the service, living arrangements, relationship status, and degree of impairment. There were also some areas of commonality between participants. Six adults with HFASD disclosed that they had received a diagnosis of ASD in adulthood, whilst three had been diagnosed in childhood and five did not say. Seven adults with HFASD said that they had received support from services outside of Leeds AIM; predominantly mainstream housing and employment support. Five adults with HFASD said that they had experienced, or continue to experience, mental health difficulties (i.e. depression, anxiety). That none of the adults with HFASD were from a black, Asian, or ethnic minority (BAME) backgrounds, and that twelve out of fourteen adults with HFASD were male, may bias and/or limit the findings.

### Data Analysis

Interview data were subject to an interpretive thematic analysis (Braun and Clarke 2006) using Nvivo. Four transcripts were initially randomly selected and analysed independently by both researchers before coming together to discuss their interpretation. This involved reading the transcripts and coding text pertinent to the research questions. Related codes were then brought together into a hierarchy as themes and subthemes. Once a consensus was reached with regard to an interpretation of the data and a thematic framework agreed, the remaining 26 transcripts were analysed by one researcher. The coding was ‘interpretive’, although the analysis did not occur in a vacuum (Braun and Clarke 2006) and was, to a lesser or greater extent, influenced by a priori knowledge from published literature and conducting interviews.

The emergent higher-order themes from the analysis (employability and education; access to information and support; reduced social isolation; health and wellbeing; and managing day-to-day) are used in this paper as subheadings to present the findings. Quotes from the interviews are presented to highlight relevant points. Quotes are attributed to individuals by a unique identifier consisting of two letters and a number. The letters denote whether the interviewee was an adult with HFASD (SU), a family member (FA), a mentor (ME) or professional (ST).

### Ethics

Leeds Beckett University provided ethical approval for the work. Given the potential vulnerability of adults with HFASD in research, additional effort was made to ensure an accessible research design (Rios et al. 2016). Potential participants were given an accessible written information sheet explaining the purpose of the project and their potential role. The service manager, as a trusted ally, also briefly spoke to adults with HFASD who expressed an interest in taking part to ensure they understood what was being asked of them. Moreover, the research was underpinned by a participatory approach. A steering group made up of the research team, service employees, and adults with HFASD guided the scope of the research and fed into the design of research instruments. The interviews with adults with HFASD were conducted by a researcher with experience of carrying out research with adults with ASD.

## Findings

The service was overwhelming described in positive terms and thought to benefit adults with HFASD, family members, and professionals and other services in the city. Engaging with the service was thought to lead to positive outcomes with regard to employability, education, volunteering, and access to support, social isolation, health and wellbeing, managing day-to-day, access to information, communication skills, and autism awareness. Being free at the point of use and aimed at people with little or no funded support, the service was thought to be “plugging a gap” (SU13) for adults with HFASD who had limited access to other forms of support. If the service were not available, it was thought that adults with HFASD and their families would be worse off.

[the service] is vital to so many people, be they autistic people or carers or even partners because it provides such an important service (SU14)

These institutions are absolutely vital if they’re serious about helping people, and helping them to live longer and helping them avoid the pitfalls of life that one would normally go down without all these kind of safety nets (SU9)

### Employability, Education and Volunteering

The service was thought to have positively affected the employment issues of adults with HFASD who used the service and helped them improve access to education and volunteering. Employability social skills training sessions and an employment peer-support group enabled adults with HFASD to feel more confident to enter the workplace. Adults with HFASD had been supported to create new, or improve existing, CVs or employment profiles.

The service facilitated opportunities to gain direct experience. A number of interviewees described progressing through different roles within the service—from service users to peer support volunteer—which had benefited their employability. Four adults with HFASD were currently employed within the organisation overseeing the service, having started out as volunteers.

The service also facilitated adults with HFASD to improve access to education by providing opportunities to consider and discuss their options in an appropriate setting. One adult with HFASD said: “if I hadn’t of been coming here…I wouldn’t of thought of going back to college” (SU10).

### Access to Information and Support

A significant outcome of the service was in enabling adults with HFASD, family members, and professionals to improve access to information and appropriate support. Interviewees described being able to gain information about a plethora of topics (i.e. housing, health, parental rights, debt management, and employment) that otherwise would not have been easily accessible.

If it wasn’t for this place I wouldn’t have known I could do a parenting plan to set ground rules on seeing my boy…it’s been a great help that way (SU8)

The significance of making information more accessible was in empowering service users to take appropriate action to address a problem or need. A family member, for example, described getting information about how her son with HFASD could transfer jobs, making the family’s impending relocation more manageable for all involved.

Those spoken to described gaining support with regard to accessing an official autism diagnosis, employment, housing, social skills training, access to computers, and legal support.

I’d probably still be struggling on how to fill in this application form because no one would have time to sit with me and explain things in more detail (SU8)

I’ve been able to speak to people who know about that stuff in a confidential and supportive environment…I’ve got the support I need (SU14)

Whilst the service primarily sought to signpost service users on to other sources of support and information, staff and volunteers also provide some short-term, direct support to users (i.e. during times of crisis) and were available to advocate on behalf of users in order for them to “get their voices heard” (SU13).

### Reduced Social Isolation

The social aspects of the service were substantial and resulted in users feeling less socially isolated. For many service users, the service provided a unique opportunity to meet and interact with “likeminded people” (SU5).

I find it very easy to get on with other autistic people…it must simply be the case that are brains are so similar that we’re on the same wave length…I can walk in here and I wouldn’t know they were autistic and start chatting…maybe that’s something you can’t do here with regular neuro-typicals (SU9)

I didn’t really have much by way of a social life or many other autistic people…to share my experiences with but since I’ve been involved in Leeds Autism AIM things have really picked up for me…I’ve been able to make more friends who are autistic…I actually have a social life (SU14)

The drop-in hubs provided opportunities for new social and, in one instance, romantic relationships for adults with HFASD. The social impact extended beyond the drop-in hub buildings as adults with HFASD described meeting up with new made friends outside of the drop-in hubs and of continuing relationships online. The service also supported users to develop their communication skills and the confidence to speak to others outside of the drop-in hubs or mentoring settings.

Since I’ve been involved in [the service] I’ve found I’ve become better at talking to people, become a better listener (SU14)

Moreover, mentoring provided opportunities for more in-depth, one-to-one interaction with a trusted person. Opportunities for volunteering within the service (i.e. as a hub volunteer or peer-mentor) provided occasions to develop more formal communication skills.

As well as facilitating new social relationships, the service was also thought to help maintain existing relationships. For example, one adult with HFASD suggested support from the service had “saved my marriage” (SU4). Another adult with HFASD had been able to maintain his relationship with his mother with whom he lived because of the support received from the service.

### Health and Wellbeing

Interviewees thought the service positively affected their health and wellbeing; to be “less miserable” and more confident (SU14). In the most extreme, the service was thought to have prevented adults with FHASD from attempting suicide or harming themselves and others.

It’s easy to say ‘I don’t know whether I’d be here now’, but I don’t…thinking about killing myself is a constant thing (SU10)

Interviewees described not having another outlet to discuss personal issues and ‘bottling up’ things that were causing them problems. The service, via the mentoring and drop-in hubs, provided opportunities to air feelings and concerns in a confidential environment; “somewhere to get it off your head” (SU1). Adults with HFASD reported that this helped reduce their anxieties and associated negative behaviours, such as aggression and/or self-harm.

Usually I don’t talk about personal things in my life that go wrong but I’m able to here, I’m happy to…I wouldn’t have had that without this place (SU5)

Another example of how the service has led to improved health outcomes is supporting adults with HFASD to develop hospital passports and health action plans.

The positive health and wellbeing benefits of the service also extended to the families of adults with HFASD who use the service. The service provided support directly to family members, whilst the indirect effect of adults with HFASD attending the drop-in hub or mentoring was to provide respite to family members.

I get three hours to myself a week. So having that extra support gives me the help I need so that I can focus on me and get my health back to where it was (FA1)

Without the autism hub to be there as that extra support…I think my mother and I would be very ill by now…it just eases the pressure tremendously on her because if there’s an issue I can come here and she’s not having to deal with it in such a way that she’s being ignored (SU9)

### Managing Day-to-Day

Whilst adults with HFASD may struggle with “everyday things that normal people take for granted” (SU4), such as paying bills and debt management, travelling around the city, being in social situations, and accessing support, the service appeared to have a positive impact on their ability to cope with such activities. The service was thought to help service users “[live] less chaotic lives” (SU13).

To go into a city centre on a hot day was unimaginable to me. Now I can do it—I may have to put my headphones on and get my head down and just walk to where I need to go—but I can if I’m with someone just stroll through and relax a bit more (SU8).

As well as supporting service users to effectively manage specific tasks, the service provided adults with HFASD with more general support about managing their autism. In reference to how they felt when their autism ‘flares up’, one interviewee said the service had helped them to develop strategies to “contain that explosion” (SU4), preventing or lessening some of the negative consequences they would have previously faced.

Through peer mentoring and interaction at the drop-in hubs, the service supported adults with HFASD to understand—and, in some cases, come to terms with—their conditions better. This enabled them to be more tolerant of themselves and other people with and without ASD.

Members of staff like **** might signpost [service users] to me and then I can talk to them and share my experience and show them that there are a lot of positives about being autistic and it is possible for us to achieve something (SU13)

The service also influenced autism awareness within other organisations by providing autism awareness training and information to other organisations. Specifically, the service raised awareness around the needs of adults with HFASD as opposed to children and their families.

## Discussion

That low-level, preventative support is needed for adults with high functioning autism spectrum disorder (HFASD) at risk of experiencing poor physical and mental health, employment, and social outcomes is championed in both academic literature and policy. This paper has explored the efficacy of a low-level support service for adults with HFASD, adding to a relatively small pool of existing research.

Providing advocacy, information, and mentoring was overwhelmingly described in positive terms and thought to benefit adults with HFASD, family members, and professionals and other services in the city. The employability, education, volunteering, accessing support and information, social isolation, health and wellbeing, and managing day-to-day of adults with HFASD were all thought to be improved through engaging with the service. At the drop-in hubs, adults with HFASD had an opportunity to feel welcome and understood, to socialise with peers, and to receive help and support that may not otherwise be accessible. The mentoring service provided a source of social interaction as well as helping mentees achieve specific outcomes, such as increased confidence and employability. The information and signposting service enabled service users to access appropriate support in a friendly and low stress environment. The service was “plugging a gap” (SU13) for adults with HFASD and their families with limited or no access to other forms of support and who would be worse off if the service was not available. In this sense, the service provided appropriate support to adults with HFASD, their families and services in the city to potentially mitigated against many of the poor health and wellbeing and social outcomes that adults with HFASD risk.

Adults with HFASD face many barriers to accessing appropriate services; navigating health and care systems can be challenging and hostile for adults with HFASD (Vogan et al. 2016). On the one hand, adults with HFASD can be at risk of not meeting the eligibility criteria for specialist ‘autism’ services (Berney 2007; Lake et al. 2014; Ward and Russell 2007). That they may also not be the right age (Ward and Russell 2007; Bruder et al. 2012; Shattuck et al. 2011; Turcotte et al. 2016) or have a late/missed ASD diagnosis (García-Villamisar and Dattilo 2010; Mawhood and Howlin 1999; Nicolaidis et al. 2013) can be further barriers to accessing support from ‘autism’ services. On the other hand, the specific needs of adults with HFASD may not be recognised or catered for within ‘mainstream’ services (Lake et al. 2014; Ward and Russell 2007; The National Autistic Society 2001). This includes the location of services in off-putting or inaccessible locations for people with HFASD (i.e. in town centres), and staff and service procedures not being accommodating to the needs of people with HFASD. A number of the adults with ASD involved in this research described being dissatisfied with the service they had received from, for example, mainstream housing, employment, or mental health services.

The low-level support provided by the service appears to have a role in supporting adults with HFASD to overcome some of these barriers. For example, whilst adults with HFASD may have difficulties approaching ‘mainstream’ services independently, housing services within the drop-in hubs provided an accessible opportunity to engage with these services and to speak in confidence to professionals in a safe/secure environment. The signposting and information element of the service made the health and social care systems easier to navigate, enabling service users to access health and social care as needed. Likewise, the advocacy service supported adults to engage with services where they may have previously had negative experiences. That the service is free at the point of use and has no eligibility criteria based on age or diagnosis appears significant for those adults with HFASD and their families with little or no other funded support (Vogan et al. 2016). In particular, the service is an opportunity for individuals who did not receive an ASD diagnosis until later in life to begin to access appropriate support.

Whilst the service was thought to improve the lives of adults with HFASD and their families, we cannot say that service users’ issues were ever completely resolved. This is not a criticism of the service per se. Firstly, the focus of the service was not on achieving specific outcomes with regard to, for example, employment or improved health—although these improvements are welcome. Rather, recognising that adults with HFASD are likely to experience complex and interrelated issues in their lives (Marriage et al. 2009; Gotham et al. 2015; Lake et al. 2014), the service aimed to “provide a little bit of help for everything” (SU8). Secondly, whilst the service provided some direct support to adults with FHASD, their families, and professionals in the city, the principal purpose was to provide advice, information and signposting. The service is comparable to the autism ‘one-stop-shops’ in Edinburgh and Glasgow (Tait et al. 2013; National Centre for Autism Studies 2006, 2007) in providing “a place, person or team, that can provide the definitive repository of information about autism support and autism services within a defined area”. Like the two sites in Scotland, the strength of the service here is as a gateway to further support. The service should be viewed, to paraphrase Tait et al. (2013), as a ‘first stop shop’ that does not principally house services but works to increase the capacity of other services and service users. However, given the ongoing austerity politics in the UK and the well-publicised associated reduction in capacity across the broader statutory and voluntary health and social care sectors, the extent to which the Leeds AIM service is able to fulfil its function to effectively direct users to further appropriate support is, at best, unknown, and, at worst, diminished.

### Limitations and Future Research

Limitations in the research design mean that the findings, whilst still valid and in line with previous research, may not be generalizable. Firstly, data collected concerns only one service, in one city in England, and so the results may not be representative of other settings within different social, political, cultural, or economic contexts. Whilst the sample was sufficient to assess broad themes relating to the impact of the service, it would be useful to explore the impact for specific groups. For example, the sample was limited in terms of ethnic diversity and it was not clear at what age all participants received an ASD diagnosis. Secondly, relying on qualitative data, individuals’ perceived experience has been placed at the centre of the research. Some quantitative data concerning the efficacy of the service was planned to be extracted from feedback forms routinely administered to service users, including adults with HFASD, family members, and professionals, as part of the service. However, only 28 feedback forms were available for use in this research. Given that the small sample prevented reliable inferential statistics being produced, this data has been omitted. Thirdly, the data was collected at one point in time rather than longitudinally to explore change.

Future research should not only look to address the identified limitations of this research but the broader deficits in the literature. That adults with HFASD can achieve positive outcomes through low-level support has been consistently demonstrated using qualitative data. Quantitative measures that policy makers and commissioners are more likely to be receptive to need to be taken (Lorenc et al. 2016). This includes longitudinal measures of individual outcomes and broader impacts of low-level interventions on health and social care. Providing insight into the economic costs and dividends of low-level support through, for example, social return on investment methodologies, are paramount, particularly in the context of austerity. Given the negative association between engaging with support services and ethnicity and socio-economic status (Shattuck et al. 2011), future research should also look to include more representative samples. If certain demographic groups are found to not engage with low-level support services than the question as to ‘why?’ needs to be answered. Moreover, given the apparent efficacy of the service on supporting people who did not receive an ASD diagnosis until adulthood, it would be beneficial to further explore these peoples’ experiences of services.

## Conclusion

An ongoing failure to address the health and social care needs of adults with high functioning autism spectrum disorder (HFASD) is likely to result in greater unmet health needs, higher use of emergency departments at times of crisis, and lower utilisation of some preventative services (Nicolaidis et al. 2013; Vogan et al. 2016). Without external services, the burden of support around adults with HFASD typically falls to families (where available), which can have negative consequences for those individuals (Engström et al. 2003). Despite the policy emphasis, there is concern about whether appropriate preventative services are accessible (Lorenc et al. 2016). This paper has explored the efficacy of an advocacy, information and mentoring service, adding to the existing literature in support of low-level interventions for adults with HFASD; there are strong indicators that the service is leading to a number of positive outcomes for adults with HFASD with little or no funded support, their families, and associated services in the city. The service delivers an ‘autism friendly’ environment where adults with HFASD are empowered to take control of the support they need. Evaluating the preventative effect of the service with regard to reducing demand on health and social care services is beyond the remit of this paper, although the service did support users to not fall under the radar or between the gaps of support structures (Baldwin and Costley 2016; The National Autistic Society 2001). Whilst such interventions would appear useful in fulfilling UK government aspirations of providing good quality health and social care for all adults with ASD, their effectiveness in the context of ongoing austerity is, at best, unknown, and, at worse, diminished.
